# Cardiovascular adaptations and pathological changes induced by spaceflight: from cellular mechanisms to organ-level impacts

**DOI:** 10.1186/s40779-024-00570-3

**Published:** 2024-09-27

**Authors:** Han Han, Hao Jia, Yi-Fan Wang, Jiang-Ping Song

**Affiliations:** Beijing Key Laboratory of Preclinical Research and Evaluation for Cardiovascular Implant Materials, Animal Experimental Centre, National Centre for Cardiovascular Disease; Department of Cardiac Surgery, Fuwai Hospital, Chinese Academy of Medical Sciences and Peking Union Medical College, Beijing, 100037 China

**Keywords:** Spaceflight, Microgravity, Space radiation, Circadian rhythm disruption, Mitochondrial dysfunction, Oxidative stress

## Abstract

The advancement in extraterrestrial exploration has highlighted the crucial need for studying how the human cardiovascular system adapts to space conditions. Human development occurs under the influence of gravity, shielded from space radiation by Earth’s magnetic field, and within an environment characterized by 24-hour day-night cycles resulting from Earth’s rotation, thus deviating from these conditions necessitates adaptive responses for survival. With upcoming manned lunar and Martian missions approaching rapidly, it is essential to understand the impact of various stressors induced by outer-space environments on cardiovascular health. This comprehensive review integrates insights from both actual space missions and simulated experiments on Earth, to analyze how microgravity, space radiation, and disrupted circadian affect cardiovascular well-being. Prolonged exposure to microgravity induces myocardial atrophy and endothelial dysfunction, which may be exacerbated by space radiation. Mitochondrial dysfunction and oxidative stress emerge as key underlying mechanisms along with disturbances in ion channel perturbations, cytoskeletal damage, and myofibril changes. Disruptions in circadian rhythms caused by factors such as microgravity, light exposure, and irregular work schedules, could further exacerbate cardiovascular issues. However, current research tends to predominantly focus on disruptions in the core clock gene, overlooking the multifactorial nature of circadian rhythm disturbances in space. Future space missions should prioritize targeted prevention strategies and early detection methods for identifying cardiovascular risks, to preserve astronaut health and ensure mission success.

## Background

Since the inception of the Mercury program more than six decades ago, over 600 astronauts have participated in space missions lasting up to 438 d. The International Space Station (ISS) has been operating within Low Earth Orbit (LEO) for more than twenty years as global space agencies aim for extended lunar and Martian expeditions. Nevertheless, deep-space missions present unique challenges compared to those experienced within LEO such as microgravity, radiation exposure, and circadian rhythm disruptions, which require thorough evaluation and management [1, 2]. Microgravity and low gravity on lunar or Martian surfaces can lead to adaptive changes in cardiovascular physiology, including myocardial atrophy and arterial wall thickening [3]. Conversely, microgravity may trigger gene expression similar to early developmental stages in cardiovascular cells, potentially facilitating myocardial regeneration [4]. Furthermore, astronauts face heightened exposure to intense space radiation comprising high-energy heavy ions [known as high charge and energy nuclei (HZE particles)] as well as protons due to lack of Earth’s magnetic shield, leading to potential vascular dysfunction along with ischemic cardiac remodeling [3]. Moreover, disturbances in the circadian rhythm caused by changes in daily schedules, lack of sleep, and other factors can collectively lead to alterations in cardiac morphology, structure, and function [5, 6].

Regardless of mission duration, evaluations of relevant physiological parameters and the implementation of risk mitigation strategies, including those associated with exercise, have been widely adopted. This review outlines the potential mechanisms driving cardiovascular structural and functional changes during spaceflight and discusses possible interventions for managing the space environment and promoting cardiovascular recovery.

## Microgravity-induced degenerations in cardiovascular structure and function

**Fig. 1:**
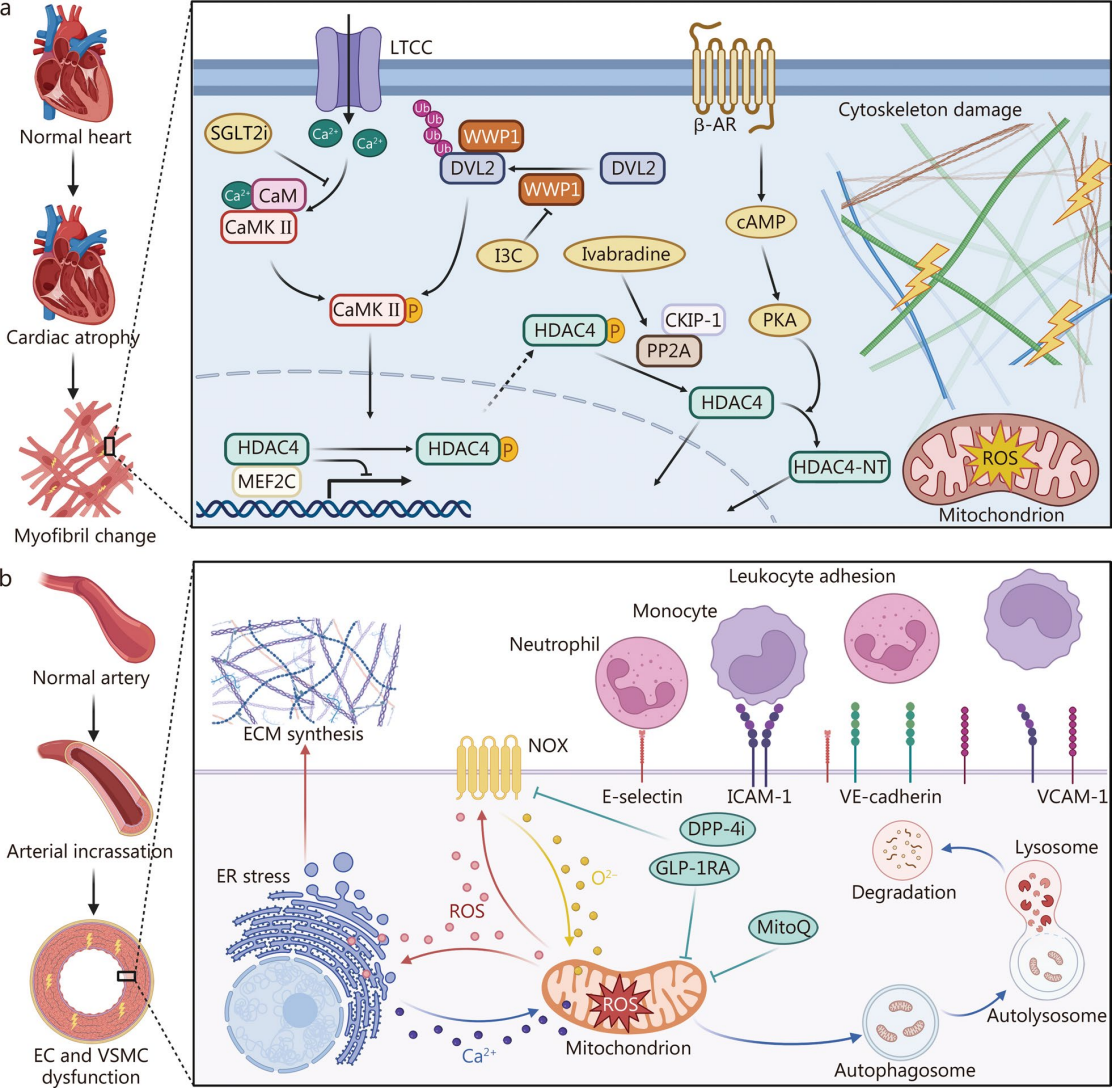
Molecular mechanisms of cardiovascular dysfunction induced by microgravity and possible countermeasures. **a** Cardiac change under microgravity. Microgravity leads to cardiac atrophy and myofibril change, with the Ca^2+^-CaMKII-HDAC4 axis playing a central role. Increased intracellular Ca.^2+^ activates CaM and CaMKII, leading to HDAC4 phosphorylation. Phosphorylated HDAC4 translocates to the cytoplasm, relieving its inhibitory effect on MEF2C and causing cardiac atrophy. Furthermore, oxidative stress is increased in mitochondria and cytoskeleton. Potential countermeasures include SGLT2 inhibitors that suppress CaMKII phosphorylation, as well as compounds like I3C and Ivabradine targeting WWP1 and PP2A, respectively. **b** Vascular alterations in microgravity. Microgravity induces arterial thickening as well as EC and VSMC dysfunction. ROS generation is a key mediator, with NOX enzymes contributing to oxidative stress. Mitochondrial dysfunction further amplifies ROS production, leading to cellular damage and mitophagy. Therapeutic strategies such as DPP-4i, GLP-1RA, and MitoQ. β-AR β-adrenergic receptor, CaM calmodulin, CaMKII calcium-calmodulin dependent protein kinase II, cAMP cyclic adenosine monophosphate, CKIP-1 casein kinase 2 interacting protein-1, DPP-4i dipeptidyl peptidase-4 inhibitor, DVL2 dishevelled segment polarity protein 2, EC endothelial cell, ECM extracellular matrix, ER endoplasmic reticulum, GLP-1RA glucagon-like peptide 1 receptor agonist, HDAC4 histone deacetylase 4, HDAC4-NT N-terminal proteolytic fragment of HDAC4, I3C indole-3-carbinol, ICAM-1 intercellular cell adhesion molecule-1, LTCC L-type calcium channels, MEF2C myocyte-specific enhancer factor 2C, MitoQ mitoquinone, NOX NADPH oxidase, PKA protein kinase A, PP2A protein phosphatase 2A, ROS reactive oxygen species, SGLT2i sodium-glucose cotransporter-2 inhibitor, VCAM-1 vascular cellular adhesion molecule-1, VSMC vascular smooth muscle cell, WWP1 WW domain-containing E3 ubiquitin protein ligase 1

Over the past few decades, a multitude of cardiovascular abnormalities associated with microgravity have been identified, including myocardial atrophy [7], systolic and diastolic dysfunction [8], and vascular dysfunction [9]. Headward fluid shifts mediated by unloading in microgravity are correlated with enhanced systolic function during short-term or early stages of long-term spaceflight [10]. However, prolonged exposure to microgravity can induce cardiac atrophy through alterations in ion channel expression or activity and downstream signaling pathways in cardiomyocytes, as well as myofiber and extracellular networks, accompanied by oxidative stress, ultimately resulting in impaired cardiac function **(**Fig. 1a**)** [11–13]. Moreover, microgravity can induce changes in vascular structure and function, and endothelial cells (ECs) are particularly sensitive to hemodynamic variations [14, 15]. Microgravity may trigger vascular wall inflammation by modulating gene expression related to cell adhesion and oxidative stress in ECs and vascular smooth muscle cells (VSMCs), leading to arterial wall thickening and stiffness that contribute to vascular dysfunction **(**Fig. 1b**)** [16, 17]. Furthermore, microgravity can enhance the expression and activity of EC nitric oxide synthase (NOS), thereby augmenting vascular relaxation [18]. Table 1 [7, 10–12, 17, 19–50] compares the potential differences in the cardiovascular system at the functional, structural, and molecular levels between short- and long-term spaceflight.

**Table 1:** Microgravity- or simulated microgravity-induced cardiovascular adaptations and remodeling

Variables	Short-term spaceflight	Long-term spaceflight	Evidence source	Reference
Functional and morphological cardiovascular adaptations and remodeling				
SV	↑ 40–46%	Unchanged or ↓ 10–15%	SF	[10, 19, 20]
LVEF	Unchanged or ↑ 6%	Unchanged or ↓ 10.5%	SF	[21, 22]
CO	↑ 18%	↓ 17–20%	SF	[10, 20]
LVEDV	↓ 7–13%	↓ 8–24%	–	[20, 22, 23]
LVESV	↓ 12%	↑ 39%	SF	[21]
LV mass	↓ 9–12%	Unchanged or ↓ 12%	SF/SMG	[7, 23, 24]
MAP	Unchanged or ↓ 6 mmHg	Unchanged or ↓ 6–10 mmHg	SF	[20, 25–27, 30]
SBP	↓ 19 mmHg	Unchanged or ↓ 8–14 mmHg	SF	[20, 25, 27, 28, 30]
DBP	Unchanged	Unchanged or ↓ 9 mmHg	SF	[20, 25, 27]
cIMT	↑ 0.06 mm	↑ 0.07 mm	SF	[29]
CAS	-	↑ 17–47%	SF	[17, 29]
fIMT	↑ 0.06 mm	↑ 0.08 mm	SF	[29]
FAS	-	↑ 20%	SF	[29]
Ultrastructural and molecular responses to short- and long-term microgravity				
Sarcomere	Less organized sarcomere arrangement demonstrating reduced sarcomere length and cTnT content	Decreased sarcomere number, loosely arranged myofibrils organized in a more longitudinal orientation	SF	[12, 38]
Cytoskeleton in CM	Disordered cytoskeleton structure	Decreased content of ACTB and ACTN4	SF	[32, 38]
Mitochondria in CM	Damaged and irregular-shaped mitochondria with higher mitochondrial volume density value and mitochondria-to-myofibril ratio	Mitochondrial recovery from swelling and disruption of cristae and denser cristae	SF/SMG	[33–35]
Ca^2+^ handling	Reduced Ca^2+^ recycling rate	Reduced Ca^2+^ recycling rate	SF	[36, 38]
HDAC4	Increased phosphorylation upregulated markers of myocardial remodeling	Increased phosphorylation augmented cardiac remodeling-related signals	SMG	[11, 31, 37]
Oxidative stress	Accumulation of ROS, mitochondrial superoxide anion, and protein carbonyls	Activation of NOX, induction of SOD2, and other transcripts involved in oxidative stress disposal	SMG/SF	[39–42]
Cytoskeleton in VSMC	Disorganized cytoskeleton with less stress fibers	–	SMG	[43]
Cytoskeleton in EC	Cells became rounded in morphology with reduction of cytoskeletal components and disruption of cytoskeleton	Redistribution of cytoskeleton from the periphery of the nucleus to the plasma membrane with reduction and alternate morphology of fibers	SMG	[44, 45]
Mitochondria in VSMC	–	Oxidative stress downregulated mitochondrial fusion and upregulated the fission, leading to more prolonged and narrow mitochondria	SMG	[46, 47]
Mitochondria in EC	Mitophagy and reduced mitochondrial content, disruption of mitochondrial cristae, and mitochondrial vacuolation	–	SMG	[48–50]

*ACTB* actin β, *ACTN4* actinin α4, *CAS* carotid artery stiffness, *cIMT* carotid intima-media thickness, *CM* cardiomyocyte, *CO* cardiac output, *cTnT*, cardiac troponin T, *DBP* diastolic blood pressure, *EC* endothelial cell, *FAS* femoral artery stiffness, *fIMT* femoral intima-media thickness, *HDAC4* histone deacetylase 4, *LV* left ventricle, *LVEDV* left ventricular end-diastolic volume, *LVEF* left ventricular ejection fraction, *LVESV* left ventricular end-systolic volume, *MAP* mean arterial pressure, *NOX* NADPH oxidase, *ROS* reactive oxygen species, *SBP* systolic blood pressure, *SF* space flight, *SMG* simulated microgravity, *SOD2* superoxide dismutase 2, *SV* stroke volume, *VSMC* vascular smooth muscle cell. “-” indicate no data

### Insights into cardiac functional changes: atrophic remodeling

Extensive degenerative changes in cardiac structure caused by microgravity have been extensively documented. Initial research conducted by the National Aeronautics and Space Administration (NASA) using chest X-rays revealed a decreased cardiothoracic ratio in 80% of Apollo astronauts [51]. However, the limitations of X-ray diagnostics, such as sensitivity to body position and respiration, and the inability to detect alterations in cardiac systolic and diastolic function, constrain their utility in evaluating cardiac health and heart failure (HF) [52]. Subsequent use of echocardiography offered a more precise assessment of left ventricle (LV) morphology and function in Skylab astronauts, demonstrating reductions in left ventricular end-diastolic dimension (LVEDD), stroke volume (SV), and mass upon return to Earth [53]. Perhonen et al. [7] confirmed similar findings using cardiovascular magnetic resonance, observing a decrease in LV mass among astronauts and bed-rest patients, suggesting that morphological atrophy of the heart occurs under the influence of microgravity.

Prolonged spaceflight has been associated with reduced cardiac systolic and diastolic functions [54]. Shibata et al. [30] utilized photoplethysmography to record 24-hour finger blood pressure waveforms and estimate SV and total cardiac output (CO), reporting a decrease in SV and 24-hour total CO after 4–6 months of spaceflight. However, these values did not differ significantly from pre-flight levels. In contrast, Herault et al. [20] observed a significant reduction in CO at 1, 3, and 5 months of flight using echocardiography and calculations based on heart rate (HR). These discrepancies may arise from different measurement methods, highlighting the necessity for more precise techniques to monitor cardiac performance. The left ventricular end-diastolic volume (LVEDV), early to late diastolic transmitral flow velocity (E/A), and early diastolic transmitral flow velocity to early diastolic mitral annular tissue velocity (E/E’) serve as crucial indicators of ventricular diastolic function [55, 56]. A prospective observational study employing tilt tests to simulate varying gravity conditions revealed an increase in LVEDV with decreasing gravity, while the E/A and E/E’ ratios remained constant [8]. Conversely, long-term spaceflight resulted in a 13% decrease in LVEDV [22], a trend also observed in a prolonged bed-rest study [57].

Studies involving rodents in spaceflight and simulated microgravity have demonstrated that, despite maintaining body weight, there is a decrease in the ratio of cardiac mass to body weight, interventricular septal mass, ventricular mass, and wall thickness [58, 59]. Histological analyses revealed a reduction in the cross-sectional area of myofibers, thinning of sarcomeres, irregular morphology, and a decreased quantity of cardiomyocyte mitochondria, indicating cardiac atrophy [34, 60]. Further investigations using tail suspension (TS) and hindlimb unloading (HU) models have confirmed that microgravity significantly diminished cardiac mass as well as the ratio of cardiac mass to body weight. It also affects ejection fraction (EF), fractional shortening (FS), LVEDD, and left ventricular end-systolic dimension (LVESD) across various species [31, 37], suggesting a consistent pattern of cardiac atrophy leading to functional deterioration. Table 2 [61–68] summarizes changes observed in animal and human models mimicking conditions during spaceflight.

**Table 2:** Animal and human models for simulating microgravity

Models	Species	Principles	Induced changes	Reference
Tail suspension and hindlimb unloading	Rodent	Remove weight-bearing loads from the hindquarters and produce a cephalic fluid shift	Muscle atrophy and bone loss, activation of the immune system, hypovolemia, tachycardia, reduced capacity to elevate peripheral vascular resistance, diminished aerobic exercise capacity, and orthostatic hypotension	[61, 62]
Head-down bed rest	Primate	Redistribution of fluid from legs to head	Headward fluid shift, the absence of work against gravity, unloading of the body’s upright weight, reduction in overall sensory stimulation, and reduced energy requirements	[63]
Dry immersion	Human	Neutral buoyancy of the human body	Fluid redistribution, vascular volume changes, and post-simulation orthostatic intolerance without medical complications such as decubitus ulcers	[64, 65]
Parabolic flight	No limit to species	Short-duration periods of free fall alternating with high-g pullout or recovery phases	Fluid shift, vagal predominance and bradycardia, baroreflex alterations, confusing vestibular signals, and visual reorientation illusion	[66–68]

#### Calcium handling and downstream signaling pathways

Kohn et al. [69] proposed that microgravity induces changes in ion channel activity by altering cell membrane fluidity. This change has been associated with calcium handling abnormalities, contributing to cardiac structural and functional impairments [70]. Echocardiography conducted after 28 d of HU treatment in mice revealed cardiac enlargement and compromised systolic function. Ventricular cardiomyocytes from these mice exhibited increased spontaneous calcium release and sarcoplasmic reticulum (SR) calcium leakage. The underlying mechanism may involve the activation of calcium-calmodulin dependent protein kinase II (CaMKII) under simulated microgravity, leading to enhanced phosphorylation of ryanodine receptor 2 (RyR2) and phospholamban, thereby disrupting normal SR calcium regulation. Additionally, parabolic flight-induced microgravity activated L-type calcium channels (LTCC) in human induced pluripotent stem cell-derived cardiomyocytes (hCMs), potentially increasing SR calcium release through calcium-induced calcium release [71]. Conversely, simulated microgravity impaired isoproterenol-stimulated L-type calcium currents in cardiomyocytes [72]. This effect was attributed to disrupted β-adrenergic receptor signaling, resulting in reduced cyclic adenosine monophosphate (cAMP) production, attenuated protein kinase A (PKA) activation, LTCC dysfunction, and decreased L-type calcium currents, ultimately leading to reduced cardiac contractility [72]. These variations may be due to rapid shifts between hypergravity and microgravity during parabolic flight, affecting LTCC activity in hCMs. These findings underscore the significant role of SR calcium handling anomalies in the decline of cardiac function induced by microgravity.

Histone deacetylase 4 (HDAC4) serves as a link between the modulation of intracellular calcium concentration and cardiac remodeling by inhibiting myocyte-specific enhancer factor 2C (MEF2C) [73]. Phosphorylation of HDAC4 at Ser467 and Ser632, which relieves the suppression of transcription factors by binding to 14-3-3 proteins and relocating to the cytoplasm, is pivotal for modulating MEF2C activity [74]. Liu et al. [11] demonstrated that simulated microgravity reduced HL-1 cell volume, increased spontaneous calcium oscillations, and elevated cytoplasmic Ca^2+^ concentrations, leading to activation of the CaMKII/HDAC4 signaling pathway and upregulation of cardiac remodeling genes such as *ANP* and *BNP*. This finding suggests that microgravity-induced cardiac atrophy potentially involves the Ca^2+^-CaMKII-HDAC4 axis. Notably, cardiovascular remodeling may exacerbate cardiac reloading. Following 28 d of HU in mice, HDAC4 phosphorylation in the LV remained unchanged; however, an increase was observed after 7 d of hindlimb reloading (HLR), consistent with changes in LVESD and LVEDD. Conversely, post-HU RV exhibited increased HDAC4 phosphorylation, which normalized after 14 d of HLR. The RV end-diastolic dimension decreased post-HU but recovered post-HLR [75], indicating differential responses in cardiac remodeling between the LV and RV.

Recent studies have revealed the crucial roles of WW domain-containing E3 ubiquitin protein ligase 1 (WWP1) and casein kinase 2 interacting protein-1 (CKIP-1) in cardiac remodeling [76, 77]. WWP1, through catalyzing K27-linked polyubiquitination, stabilizes dishevelled segment polarity protein 2 (DVL2), thereby enhancing the CaMKII-HDAC4-MEF2C axis and promoting cardiac remodeling [78]. Simulated microgravity induced by TS resulted in elevated expression of WWP1 and DVL2. *WWP1* knockout alleviated cardiomyocyte volume reduction, cardiac atrophy, and functional decline [37]. By facilitating the interaction between the catalytic subunit of protein phosphatase 2A (PP2A) and HDAC4, CKIP-1 promotes HDAC4 dephosphorylation and nuclear retention, thereby dose-dependently inhibiting MEF2C transcriptional activity and influencing cardiac remodeling [79]. In murine hearts subjected to microgravity simulations, decreased CKIP-1 expression was observed, while CKIP-1 overexpression countered cardiomyocyte volume reduction and cardiac atrophy [31]. These findings suggest that targeting the Ca^2+^-CaMKII-HDAC4-MEF2C pathway and its associated regulatory proteins may hold promise as a therapeutic approach for addressing microgravity-induced cardiac atrophy and dysfunction.

#### Assembly and degradation of contractile proteins

In comparison to specimens subjected to ground control conditions, Drosophila exposed to 30 d of spaceflight exhibited significant reductions in end-diastolic diameter and FS, leading to decreased CO. Microgravity exposure resulted in the reorientation of cardiac myofibrils from circumferential to longitudinal orientation, with a more relaxed arrangement, increased interstitial spacing, and a notable decrease in sarcomere count [12]. Simultaneous downregulation of human actin homologs, such as *Actin88F* (*hACTB*) and *Actin79b* (*hACTA/B*), along with upregulation of genes encoding proteasome subunits, suggests compromised protein folding or increased degradation in the Drosophila heart [12]. Similar findings were observed in mouse studies, indicating downregulation of genes crucial for cardiac actin stabilization, polymerization, actomyosin assembly, and focal adhesion, implying alterations in the function and assembly of myocardial proteins [80]. Interestingly, species-specific differences were noted in the extracellular matrix (ECM) changes. Drosophila exhibited a significant reduction in the collagen network associated with myofibers alongside downregulation expression of genes involved in collagen cross-linking and ECM formation, such as *plod* and *lox*, as well as genes related to collagen degradation like matrix metalloproteinase 1 (*MMP1*) associated with myofibril remodeling. In contrast, mice displayed limited changes related to ECM gene transcription except for the downregulation of *Col4a5* and the upregulation of *Itgb1*, highlighting species-specific responses within the myofibril-associated network under microgravity conditions. Overall, these studies underscore the role of microgravity in cardiac remodeling through gene regulation affecting myofibril assembly or stabilization while influencing protein degradation processes that may lead to cardiac atrophy.

#### Oxidative stress and cytoskeleton collateral damage

Mitochondrial stress is a key factor contributing to the physiological changes observed during spaceflight [13]. Microgravity-induced oxidative stress in cardiomyocytes leads to cardiac dysfunction. Rats exposed to microgravity for 6 d exhibited a notable increase in both the activity and mRNA levels of myocardial malate dehydrogenase (MDH), as well as elevated mRNA and protein levels of subunits III, IV, and VIc of mitochondrial cytochrome c oxidase (CytOx). Despite this observation, overall myocardial CytOx activity remained unaltered [81]. Conversely, skeletal muscle did not exhibit changes in MDH or CytOx expression but displayed a 41% reduction in CytOx activity. Transcriptomic analysis conducted on hCMs after spaceflight revealed significant enrichment of genes related to mitochondrial metabolic pathways, electron transport chain function, mitochondrial respiratory chain activities, and myocardial contraction [36], indicating that the adaptive response within mitochondria following short-term exposure to microgravity was more prominent in myocardium compared to skeletal muscle. Nevertheless, the upregulation of stress-related pathways and redox-related genes in hCMs did not correspond to the upregulation of aerobic respiration-related pathways or genes [36, 82], implying oxidative damage to cardiomyocytes. Acharya et al. [82] reported that microgravity led to an increase in reactive oxygen species (ROS) in hCMs, resulting in loss of mitochondrial membrane potential and diminished ATP synthesis. Following 48 h of exposure to microgravity, cardiomyocytes showed impaired calcium transients, reduced LTCC function, and decreased expression of RyR2 and phospholamban, leading to a significant slowing contraction and relaxation speeds [82]. These findings highlight a strong link between microgravity-induced oxidative stress and subsequent cardiac functional impairment.

Oxidative stress also impacts the cytoskeleton of cardiomyocytes. hCMs exposed to microgravity exhibited characteristic changes in the cell membrane, including stress fibers and caveolae, as well as increased ROS levels [82]. H9C2 cells subjected to simulated microgravity for 24 to 48 h showed a notable increase in cell height. After 96 h, there was a significant increase in the average length of microfilaments despite stable expression of cytoskeletal components such as β-tubulin and β-actin [39]. These structural alterations were concurrent with heightened intracellular ROS, mitochondrial superoxide anion levels, and protein carbonylation. Interestingly, treatment with the antioxidant N-acetylcysteine (NAC) mitigated the effects of microgravity on cardiomyocyte morphology in H9C2 cells. This suggests that while microgravity may not cause irreversible damage to the cytoskeleton, it disrupts oxidative balance and potentially hinders cellular proliferation.

Liang et al. [42] used a murine TS model to investigate how calpain activation and oxidative stress contribute to diminishing cardiomyocyte volume, heart mass, and myocardial function. Their study on cultured mouse cardiomyocytes exposed to simulated microgravity demonstrated that calpain facilitated the phosphorylation of p47 via the extracellular regulated protein kinase 1 and 2 (ERK1/2) and p38 pathways, triggering NADPH oxidase (NOX) activation with resultant myocardial abnormalities [42]. These findings suggest the potential use of calpain inhibitors as a therapeutic approach for alleviating microgravity-induced cardiac issues [41]. Notably, peak ERK1/2 and p38 activity induced by simulated microgravity occurred on day 14 but diminished on day 28. Despite inhibiting ERK1/2 and p38 activity, incomplete blockade of p47 phosphorylation implies that these kinases act as early mediators of NOX induction, while other mechanisms might drive NOX stimulation leading to cardiac atrophy during later stages of microgravity exposure [42].

### Insights into vascular alterations: EC and VMSC dysfunction

The effects of microgravity on vascular structure and function during spaceflight are significant. The transition to space results in a shift of bodily fluids towards the head, leading to changes in peripheral and shear stress, which in turn impact blood pressure and flow, respectively [54]. Studies conducted by NASA and the European Space Agency have revealed an increase in intima-media thickness (IMT) and arterial stiffness after long-duration ISS missions [17, 29, 83], aligning with the prediction that the walls of arteries above the heart would thicken in response to elevated pressure [3]. However, measurements of femoral artery IMT in astronauts have shown an unexpected increase, contradicting predictions of atrophy below the heart [29], suggesting additional factors at play beyond microgravity during spaceflight. Both humans and rodents have demonstrated a time-dependent rise in IMT, media cross-sectional area, and arterial stiffness under simulated microgravity [84, 85]. Given that IMT and carotid artery stiffness are indicators of cardiovascular disease risk, these findings imply that spaceflight-associated microgravity may heighten astronauts’ susceptibility to cardiovascular events [86, 87]. Furthermore, Li et al. [88] conducted a study on the impact of the microgravity-induced headward fluid shift on the pulmonary circulation. They observed hypertrophy of the VMSCs in the media, arterial wall thickening and stenosis, as well as vascular hyperplasia in the pulmonary arterioles. The microvasculature displayed changes similar to those of the arteriola, including varying degrees of broadening, uneven intimal thickness, hyperplasia, degeneration, and detachment of the ECs, along with VSMC hypertrophy. The remodeling of the pulmonary vascular structure resulted in increased vascular resistance, potentially heightening susceptibility to pulmonary vascular disease such as pulmonary arterial hypertension, and exacerbating RV dysfunction and remodeling in astronauts [89].

#### Endothelial inflammation and metabolic disorder

The endothelium, essential for maintaining vascular homeostasis, releases vasoactive factors, inhibits platelet aggregation and leukocyte adhesion, and modulates VSMC proliferation [90]. ECs act as mechanoreceptors and are highly sensitive to changes in blood fluid dynamics [14, 15]. Versari et al. [16] observed differential expression of 1023 genes involved in cell adhesion, oxidative phosphorylation, stress response, cell cycle, and apoptosis in human umbilical vein endothelial cells (HUVECs) exposed to microgravity for 10 d. Moreover, exposure to simulated microgravity for 24 h increased E-selectin expression in HUVECs [91]. Upon microgravity stimulation, intercellular cell adhesion molecule-1^low^ (ICAM-1^low^) ECs upregulated ICAM-1, vascular cellular adhesion molecule-1 (VCAM-1), and VE-cadherin, and these effects were further enhanced by tumor necrosis factor stimulation [91]. E-selectin, ICAM-1, and VCAM-1 are pivotal for slowing leukocyte rolling and promoting leukocyte adhesion and inflammation [92], while VE-cadherin is crucial for maintaining vascular integrity [93]. These findings suggest that microgravity may exacerbate inflammation by modulating EC adhesion molecules, and pre-existing inflammatory conditions could intensify this effect [94].

Microgravity induces mitochondrial autophagy in ECs to counteract the metabolic challenges of mechanical unloading. Research linking the cytoskeleton to mitochondrial autophagy has shown that treating HUVECs with cytochalasin D leads to actin cytoskeletal and mitochondrial changes similar to those observed under microgravity [49]. Microgravity exposure increased the expression of B-cell lymphoma 2/adenovirus E1B 19 kD protein-interacting protein 3 (BNIP3), a marker of mitochondrial autophagy, while decreasing mitochondrial content, oxygen consumption, and maximal respiratory capacity. These effects were partially reversed by chloroquine, an autophagy inhibitor [49]. Furthermore, microgravity disrupted endoplasmic reticulum (ER) protein homeostasis in HUVECs, leading to ER stress and Ca^2+^ transfer to mitochondria, resulting in mitochondrial Ca^2+^ overload, reduction in membrane potential, mitochondrial fission, and activation of the phosphatase and tensin homolog (PTEN)-induced putative kinase 1 (PINK1)-Parkin pathway, further inducing mitochondrial autophagy [50]. Mitochondrial dysfunction increased ROS production and activated the NOD-like receptor thermal protein domain associated protein 3 (NLRP3) inflammasome; these processes can be attenuated by MitoTempo treatment and *PINK1* knockout [50]. Thus, microgravity may promote mitochondrial autophagy through disruption of the cytoskeletal and ER stress mechanisms that facilitate cellular adaptation for maintaining essential functions and ensuring survival [66]. Additionally, exposure to microgravity could induce endothelial inflammation and apoptosis via ER stress [95]. Microgravity upregulated ER stress-related proteins such as C/EBP-homologous protein (CHOP) and glucose-regulated protein 78, and pro-inflammatory cytokines including interleukin (IL)-6, tumor necrosis factor-α, IL-8, and IL-1β, followed by activation of inducible NOS (iNOS)/NO-nuclear factor kappa-B (NF-κB) and NLRP3 signaling pathways, resulting in endothelial inflammation and apoptosis. This ER stress was confirmed using inhibitors for iNOS/NO, NF-κB, or small interfering RNA targeting NLRP3 [95].

#### NOS-dependent vascular dysfunction

Nitric oxide (NO) plays a crucial role in maintaining vascular health. However, exposure to microgravity may lead to endothelial dysfunction related to NO [18]. Simulated microgravity exposure resulted in a significant upregulation of endothelial NOS (eNOS) and iNOS in rat carotid and thoracic arteries, while the expression levels decreased in the mesenteric artery, indicating tissue-specific responses [96]. Sangha et al. [97] reported that 20 d of simulated microgravity reduced vasoconstriction response to norepinephrine. This effect was reversed by endothelial removal or iNOS inhibition, suggesting that enhanced iNOS expression or activity under microgravity might augment arterial dilation dependent on the endothelium. Activation of eNOS may also contribute to hyporesponsiveness under microgravity [98]. Specifically, phosphorylation at Ser1177 increased, and phosphorylation at Ser495 decreased after 3 d of simulated microgravity, resulting in increased eNOS activity, enhanced downstream NO and cGMP signaling along with heat shock protein 90, subsequently leading to decreased vasoconstriction [98]. In contrast, one study has shown that HU can induce enhanced vasoconstriction and reduced endothelium-dependent relaxation through angiotensin II type 1 receptor-mediated decrease in iNOS content [99]. These discrepancies may be attributed to variations in arterial locations: the former two studies employed femoral and aortic arteries below or parallel to the heart, whereas the latter study focused on basilar and common carotid arteries above the heart. The impact of microgravity on pulmonary circulation has been elucidated. Sun et al. [100] observed that the contraction of the pulmonary artery in response to phenylephrine was markedly diminished, while vasodilation in response to acetylcholine was significantly enhanced. These distinct responses could be eliminated by endothelium removal. Nyhan et al. [101] further investigated the reactions of pulmonary arterial endothelium to microgravity at the molecular level. eNOS and soluble guanylyl cyclase expressions were significantly increased in both pulmonary artery and lung tissue from HU rats, which could account for varied responses to vasoactive agents. These findings imply the remodeling of pulmonary vasculature might affect right ventricular function, potentially leading to right heart strain or failure if significant changes occur.

#### Mitochondrial stress and phenotypic transformation of VSMCs

Microgravity has been associated with mitochondrial dysfunction, leading to oxidative stress in VSMCs. Research on the correlation between mitochondrial dysfunction and NOX has revealed that exposure to simulated microgravity for 4 weeks resulted in elevated levels of mitochondrial ROS, decreased mitochondrial membrane potential, increased opening of the mitochondrial permeability transition pore, and altered respiratory control ratio in cerebral artery VSMCs. These changes were accompanied by a significant reduction in the expression of mitochondrial superoxide dismutase and glutathione peroxidase-1. The administration of apocynin, a NOX inhibitor, ameliorated these effects [102]. Zhang et al. [103] reported that simulated microgravity enhanced superoxide production and NOX activity in cerebral artery VSMCs, leading to a significant upregulation of NOX2 and NOX4. Treatment with the mitochondrial antioxidant MitoTempo mitigated these conditions. These results suggest an interplay between NOX and mitochondria, wherein mitochondria regulate NOX expression and activity under microgravity, while conversely, NOX influences mitochondrial function by modulating superoxide radicals. However, mesenteric artery VSMCs did not respond to microgravity, indicating tissue-specific variability in microgravity-induced mitochondrial dysfunction and oxidative stress [102, 103].

Moreover, oxidative stress not only impacts mitochondrial function but also influences the ER, leading to phenotypic changes in VSMCs [46]. The interaction between mitochondrial oxidative stress and ER stress activates the protein kinase RNA-like endoplasmic reticulum kinase—eukaryotic initiation factor 2α—activating transcription factor 4—CHOP and phosphatidylinositide 3-kinase/protein kinase B (Akt)/mammalian target of rapamycin signaling pathways, promoting the transition from a contractile phenotype to a synthetic phenotype in cerebral artery VSMCs. This phenotypic change was characterized by a decrease in the expression of contractile markers and an increase in the expression of osteopontin and elastin. Osteopontin, which is abundant in atherosclerosis (AS) plaques and associated with macrophages and foam cells, impedes EC migration and proliferation, thereby suppressing re-endothelialization and promoting medial VSMC migration as well as AS development [104]. Elastin, linked to AS through the action of elastase and the generation of pro-oxidative elastin-derived peptides, further contributes to the oxidation of low-density lipoprotein and vascular wall calcification [105]. These findings indicate that microgravity may induce VSMC dedifferentiation and exacerbate vascular inflammation, potentially leading to alterations in arterial wall structure.

### Potential countermeasures targeting microgravity

Changes in Ca^2+^-dependent signaling, mitochondrial dysfunction, and ER stress have been identified as significant cardiovascular risk factors for astronauts on extended space missions. The pathophysiological parallels between diabetic cardiomyopathy, characterized by systemic insulin resistance and cardiomyocyte contractile dysfunction, and microgravity-induced cardiovascular alterations, particularly with respect to abnormal Ca^2+^ handling and mitochondrial and ER stress, are noteworthy [106]. These similarities, coupled with the observed elevation of astronauts’ insulin resistance index during prolonged spaceflight [17], underscore the potential of repurposing anti-diabetic medications or other drugs to target the Ca^2+^-CaMKII-HDAC4-MEF2C signaling pathway and its regulatory proteins, mitigating mitochondrial and ER stress, thereby enhancing cardiovascular function in space.

Empagliflozin (a selective sodium-glucose cotransporter-2 (SGLT2) inhibitor) improves Ca^2+^ handling by reducing CaMKII autophosphorylation and CaMKII-dependent RyR2 phosphorylation, as well as inhibiting O-GlcNAcylation in cardiomyocytes [107]. Similarly, teneligliptin (a dipeptidyl peptidase-4 (DPP-4) inhibitor) and exendin-4 [glucagon-like peptide 1 receptor agonist (GLP-1RA)], in combination with endurance training, significantly decrease NOX4-mediated oxidative stress and HDAC4 phosphorylation and expression, thereby enhancing cardiac remodeling [108, 109]. Ivabradine (an I_f_ current antagonist) binds to and enhances the catalytic subunit of PP2A, promoting HDAC4 dephosphorylation and improving cardiac remodeling [79, 110]. Indole-3-carbinol (a natural WWP1 inhibitor found in cruciferous vegetables) may attenuate the stabilizing effect of WWP1 on DVL2, resulting in downregulation of the Ca^2+^-CaMKII-HDAC4-MEF2C axis [78, 111]. For ECs and VSMCs, DPP-4 inhibitors and GLP-1RAs can reduce ROS production and downregulate NF-κB, NOX, and pro-inflammatory cytokine expression. This helps to maintain eNOS phosphorylation, thus reducing EC senescence and apoptosis while inhibiting VSMC migration and promoting apoptosis [112, 113]. Additionally, AKB-9778 (a vascular endothelial protein tyrosine phosphatase inhibitor) and MitoQ (a mitochondrial antioxidant), improve endothelial function and decrease vascular stiffness by preventing eNOS dephosphorylation, increasing NO production in diabetic patients, as well as reducing mitochondrial ROS generation in elderly individuals [114, 115].

Exercise regimens customized to maintain physical fitness demonstrate the potential to mitigate the cardiac effects of prolonged microgravity. However, it is important to note that the type, duration, and intensity may not completely prevent structural and functional cardiovascular changes, such as cardiac dysfunction, arterial thickening, and stiffness [9, 17, 30]. In diabetic cardiomyopathy, exercise is crucial for combating cardiovascular impairments. The N-terminal proteolytic fragment of HDAC4 (HDAC4-NT) exerts a cardioprotective effect during aerobic activities like running by elevating HDAC4-NT levels in cardiomyocytes through the cAMP-PKA pathway, thereby repressing MEF2C transcription and improving cardiac function [116]. Intriguingly, running also activates myocardial AMP-activated protein kinase, leading to HDAC4 phosphorylation and subsequent MEF2A-mediated repression of glucose transporter 1 expression. This facilitates cardiac function and glucose metabolism in ischemic HF [117]. Aerobic exercise further attenuates inflammation and oxidative stress in vascular tissues, while enhancing the bioavailability of antioxidant enzymes and NO. It also improves endothelial function and arterial rigidity [118]. Conversely, resistance exercise may compromise endothelial function due to sustained elevations in blood pressure [119]. Nevertheless, a combination of aerobic and resistance exercise appears to be beneficial for enhancing endothelial function, particularly for individuals with type II diabetes [120]. Notably, individualized exercise prescriptions can be tailored for astronauts, with high-intensity and low-repetition resistance exercise effectively circumventing vascular damage, although the underlying mechanisms remain unclear [119]. Blood flow restriction (BFR) training presents a compelling alternative to conventional resistance training by reducing joint stress while yielding comparable gains in muscle strength [121]. However, further exploration to balance the benefits against possible risks associated with BFR training such as alterations in venous wall tension and compliance due to inflatable cuffs applied to the proximal ends of limbs.

## Space radiation-induced changes in cardiovascular structure and function

**Fig. 2:**
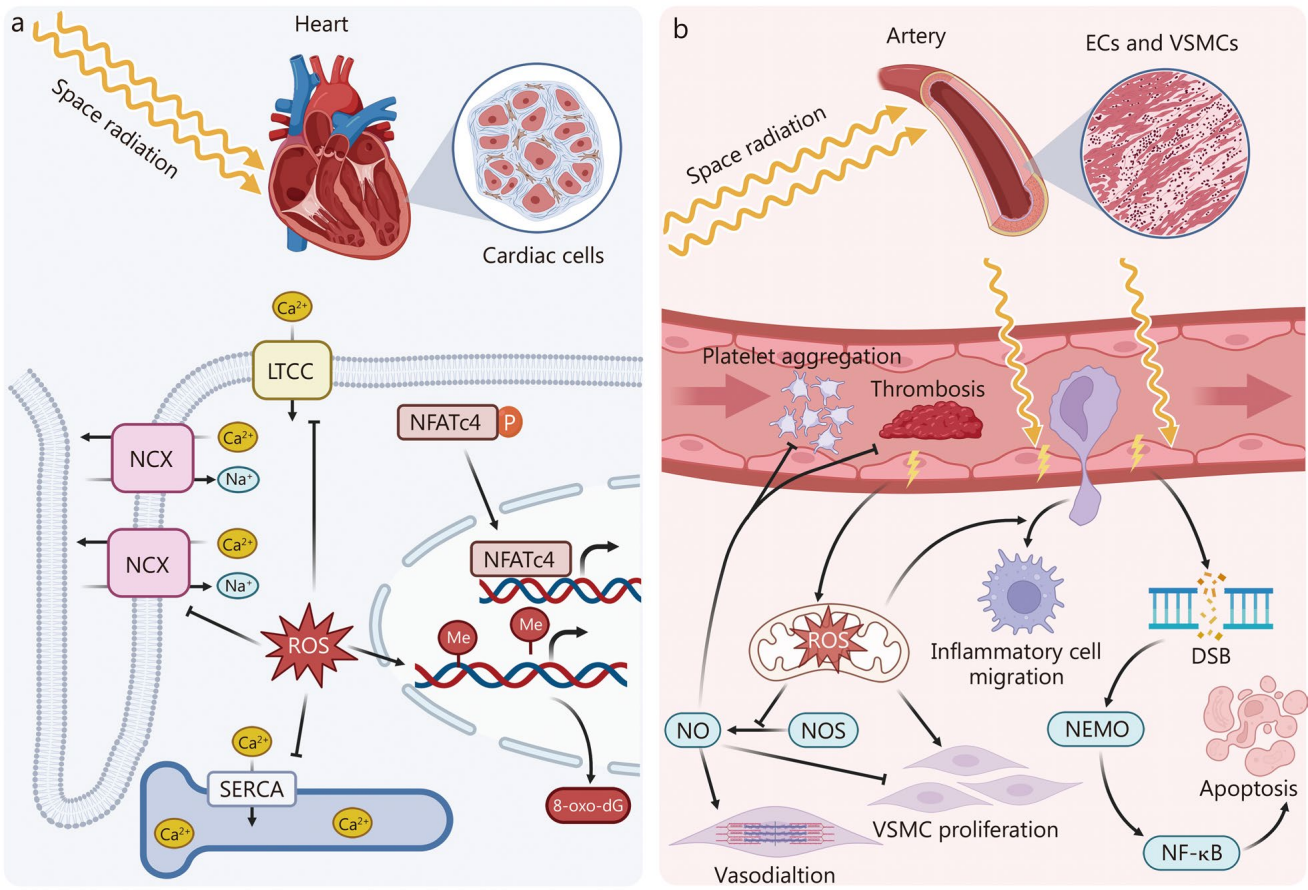
Space radiation-mediated dysfunction of cardiomyocytes, ECs, and VSMCs.** a** Cardiac response to space radiation. Exposure to space radiation leads to the generation of ROS, which disrupts calcium homeostasis in cardiac cells by inhibiting LTCC and NCX in the cell membrane of cardiomyocytes as well as SERCA in the membrane of sarcoplasmic reticulum (SR). ROS can also cause DNA damage and oxidative modifications such as 8-oxo-dG, contributing to cardiomyocyte damage and dysfunction. Space radiation induces dephosphorylation of the transcription factor NFATc4, leading to its translocation into the cardiomyocyte nucleus, along with DNA demethylation that potentially activates gene expression related to cardiac remodeling. **b** Vascular response to space radiation. Space radiation induces oxidative stress in the vascular system, promoting ROS production and resulting in EC and VSMC dysfunction. Increased ROS leads to inflammatory cell migration and disruption of the NOS-NO pathway. The NOS-NO pathway plays an important role in suppressing VSMC proliferation, platelet aggregation, and thrombosis as well as promoting vascular relaxation. Furthermore, DNA DSB induced by radiation activates NF-κB signaling via NEMO, leading to inflammation and apoptosis that further exacerbate vascular integrity damage. DSB double-strand break, EC endothelial cell, LTCC L-type calcium channel, Me methyl, NCX sodium-calcium exchanger, NEMO nuclear factor kappa-B essential modulator, NF-κB nuclear factor kappa-B, NFATc4 nuclear factor of activated T-cell c4, NO nitric oxide, NOS nitric oxide synthase, SERCA SR Ca^2+^-ATPase, ROS reactive oxygen species, VSMC vascular smooth muscle cell, 8-oxo-dG 8-oxo-deoxy-guanosine

Space radiation, particularly HZE particles, poses a significant risk for cardiovascular structural and functional changes **(**Fig. 2**)**. Its biological impact is generally more pronounced than that of terrestrial gamma rays and X-rays, highlighting the need for comprehensive protective strategies during long-duration spaceflight [122].

### Insights into cardiac diastolic dysfunction: oxidative stress and gene expression

Radiation is intricately associated with cardiac dysfunction, as research has revealed the varying impacts of different types and doses of radiation on cardiac health. In male mice, exposure to ^16^O resulted in significant declines in EF and FS at 3- and 7-months post-radiation, accompanied by the deposition of α-smooth muscle actin and 75-kD type III collagen within the LV, indicating compromised systolic function and early-stage cardiac remodeling [123]. Conversely, female mice did not exhibit functional changes despite similar biomarker deposition, suggesting a potential sex-related difference in the cardiac response to space radiation [124]. Brojakowska et al. [125] further explored the cardiac ramifications of exposure to simplified galactic cosmic radiation, which included 5 ions (^1^H, ^28^Si, ^4^He, ^16^O, ^56^Fe), at doses of 0.5 Gy and 1 Gy in male mice. Within one year, all mice displayed a significant reduction in EF. At 660 d, those exposed to 0.5 Gy showed no change in EF but demonstrated a notable decrease in LV mass and elevated levels of transforming growth factor-β1, monocyte chemoattractant protein 1, MMP9, and β-myosin heavy chain, indicating increased fibrosis, inflammation, and hypertrophy. Mice exposed to proton radiation initially experienced myocardial hypertrophy and improved systolic function, which subsequently transitioned to cardiac atrophy and fibrosis, resulting in diastolic dysfunction [123]. These findings emphasize the necessity for further exploration into the early effects of space radiation and confirm its role in inducing late-stage diastolic dysfunction, as well as cardiac atrophy, and myocardial fibrosis.

NASA’s investigation into the cardiovascular effects of space radiation revealed that exposure to ^1^H and ^56^Fe significantly increased the phosphorylation of H2A histone family member X, a marker for DNA double-strand break, by 2- to fivefold within 2 to 24 h, with detectable levels persisting at 28 d. Concurrently, CD68 (a monocyte-macrophage marker) and 8-oxo-deoxy-guanosine (8-oxo-dG; an indicator of DNA oxidative damage) were elevated in cardiac tissues [126]. The presence of 8-oxo-dG, a byproduct of ROS-induced damage, indicates oxidative stress in failing hearts [127]. Excessive ROS disrupts cardiomyocyte electrophysiology and contraction by impairing critical proteins involved in excitation–contraction coupling, including LTCCs, sodium channels, potassium channels, and sodium-calcium exchangers (NCXs). ROS also inhibits SR Ca^2+^-ATPase (SERCA), reduces the sensitivity of sarcomeres to Ca^2+^, and causes energy deficits by targeting metabolic proteins. Furthermore, the generation of ROS-induced DNA damage activates the protein kinase ataxia-telangiectasia mutated, thereby facilitating DNA repair and inhibiting cell cycle-related pathways [128, 129]. Oxidative stress induces an inflammatory response in the heart and recruits monocytes/macrophages to promote timely tissue repair through the synthesis of endogenous lipid mediators, thus preventing chronic inflammation and compromised cardiac repair [130]. The combined effects of microgravity and space radiation may exacerbate oxidative stress, potentially amplifying detrimental impacts on cardiac structure and function.

Subsequent observations revealed an initial increase in NCX and SERCA2a expression at 1-month post-radiation, followed by declines at 3 months. At 10 months, NCX levels increased again in mice exposed to ^56^Fe, while phosphorylation of the nuclear factor of activated T-cell c4 (NFATc4) progressively decreased from 1 to 10 months [126]. The upregulation of NCX, a hallmark of HF, compensates for compromised contractility; however, NCX dysfunction has been found in ventricular cardiomyocytes of failing hearts [131]. Additionally, SERCA2a, crucial for calcium handling and systolic function enhancement, is downregulated in HF [132]. NFATc4, a regulator of cardiac hypertrophy, remains highly phosphorylated and inactive under baseline conditions, but its dephosphorylation activates itself and contributes to cardiac hypertrophy [133, 134]. Space radiation also impacts cardiac health through decreased DNA methylation levels, particularly within repetitive genetic sequences [135, 136]. Exposure to ^56^Fe, ^16^O, and ^1^H has been shown to reduce the methylation and expression of long interspersed nuclear elements 1 [135, 136]. Subsequent investigations have indicated that space radiation might impair DNA methylation by modifying components related to one-carbon unit and methionine metabolism pathways, consistent with radiation-induced hypomethylation [136]. Importantly, the combined effects of microgravity and space radiation exacerbate this reduction in the potential for DNA methylations [137]. Previous research has established a correlation between reduced promoter region methylation for *ANP* and *BNP* with their activation states, indicating a link between radiation-induced hypomethylation and cardiac dysfunction [138].

### Insights into arterial remodeling: homeostasis disruption and inflammation

Space radiation has a significant impact on arterial structure and function. Mice exposed to ^56^Fe exhibited intimal thickening in the carotid artery as an indication of potential arterial wall damage associated with compromised vasodilation [139]. ECs and VSMCs exposed to radiation experienced apoptosis, oxidative stress, mitochondrial dysfunction, and cellular senescence [140]. EC apoptosis precedes vascular injury and reduced vascular density, with high linear energy transfer (LET) particles posing a greater risk of ischemic damage compared to low LET particles [141]. Vascular tissues showed significant oxidative stress following radiation, contributing to mitochondrial dysfunction and endothelial impairment [140]. Exposure to neutrons and ^60^Co also demonstrated evidence of mitochondrial damage [142]. The bioavailability and signaling of NO are essential for endothelial homeostasis. However, oxidative stress disrupts NO pathways, leading to endothelial dysfunction [143]. Exposure to HZE particles increased ROS production in the aorta, impairing NO signaling and endothelium-dependent vasodilation [144]. HUVECs exposed to ^137^Cs exhibited premature senescence through double-strand break/NF-κB essential modulator/NF-κB signaling pathways, contributing to AS development [145]. Activation of inflammatory signaling and diminished vascular endothelium integrity promote the adhesion and transmigration of inflammatory cells [146]. The impact of radiation on inflammation during plaque formation includes increased EC permeability and the facilitation of inflammatory cells and VSMC migration or proliferation [147, 148], potentially accelerating age-dependent AS [139]. Moreover, ApoE^−/−^ mice exposed to ionizing radiation at doses of 10 Gy or ^56^Fe exhibited accelerated plaque formation [139, 149].

### Potential countermeasures to space radiation

In the field of radiation therapy, there is an ongoing search for pharmacological agents to mitigate cardiac toxicity. Current strategies focus on identifying and closely monitoring high-risk individuals, coupled with managing resulting complications symptomatically. Nonetheless, dietary regimens and nutritional supplementation have emerged as potential ways to reduce oxidative stress and inflammation, offering hope for astronaut health in the context of radiation protection. The Mediterranean diet, which is rich in monounsaturated fatty acids, has been linked to reduced arterial stenosis and improved endothelial function [150]. Additionally, a low-fat diet has proven effective in the primary prevention of cardiovascular disease [151]. Despite its vascular health benefits, caloric restriction is not recommended for astronauts due to the risk of skeletal and muscular atrophy during spaceflight [152]. It is essential to strategically incorporate functional foods and antioxidant micronutrients for space missions. A summary of promising nutritional supplements can be found in Table 3 [153–159].

**Table 3:** Potential anti-oxidants with recommended dosage

Nutraceuticals	Main findings	Dosage	Reference
Ω-3	(1) Ω-3 exerted an anti-inflammatory effect attributed to the synthesis of metabolites (2) Ω-3 reduced arrhythmias via direct inhibition of sarcolemmal ion channels	1–4 g/d of eicosapentaenoic acid + docosahexaenoic acid	[153]
Vitamin C	(1) Vitamin C, as an antioxidant, improves endothelial dysfunction (2) Vitamin C might significantly decrease the incidence of atrial fibrillation	200 mg/d	[154]
Vitamin E	(1) Vitamin E was an antioxidant with lipoperoxyl radical-scavenging activities, attenuating AS (2) Vitamin E had anti-inflammatory and anti-atherothrombotic effects by modulating platelet and clotting systems	50 mg/d	[155]
Nitrate	Dietary nitrate restored the tissue levels of bioactive nitrogen oxides and reduced oxidative stress markers	5–9 mmol/d of NO_3_^−^	[156]
Lycopene	(1) Lycopene was an antioxidant with radioprotective properties via free-radical scavenging activity (2) Lycopene exerted anti-inflammatory, antiatherogenic, and antiplatelet effects and improved endothelial function (3) Lycopene could attach to plasma low-density lipoprotein cholesterol, preventing lipid peroxidation	15–75 mg/d	[157]
NR	As a NAD^+^ precursor vitamin, NR improves mitochondrial dysfunction and metabolic syndrome	250–1000 mg/d	[158]
Oxypurinol	Oxypurinol presented antioxidant properties by reducing the production of ROS derived from purine metabolism	≤ 300 mg/d or 2400 mg only	[159]

*Ω-3* omega-3 polyunsaturated fatty acids, *AS* atherosclerosis, *NR* nicotinamide riboside, *NAD*^+^ nicotinamide adenine dinucleotide, *ROS* reactive oxygen species

## Circadian rhythm disruption-related changes in cardiovascular structure and function

The circadian system is regulated by a central pacemaker clock located in the suprachiasmatic nuclei of the hypothalamus, which synchronizes physiological rhythms with environmental cues and coordinates peripheral clocks. At the molecular level, circadian oscillations are governed by a transcription-translation feedback loop involving 4 core clock gene families: *Bmal1*, *Clock*, *Period* (*Per*), and *Cryptochrome* (*Cry*), all exhibiting near-24-hour rhythmicity. CLOCK and brain and muscle arnt-like 1 (BMAL1), as part of the positive limb of the loop, stimulate the transcription and translation of *Per* and *Cry*, leading to the accumulation of PER and CRY proteins. These proteins form complexes through dimerization that represses the transcription of *Clock* and *Bmal1* [160]. Additional genes, such as *Rev-erb-α/β*, modulate this feedback loop, contributing to the robustness and precision of the circadian rhythm [161].

**Fig. 3:**
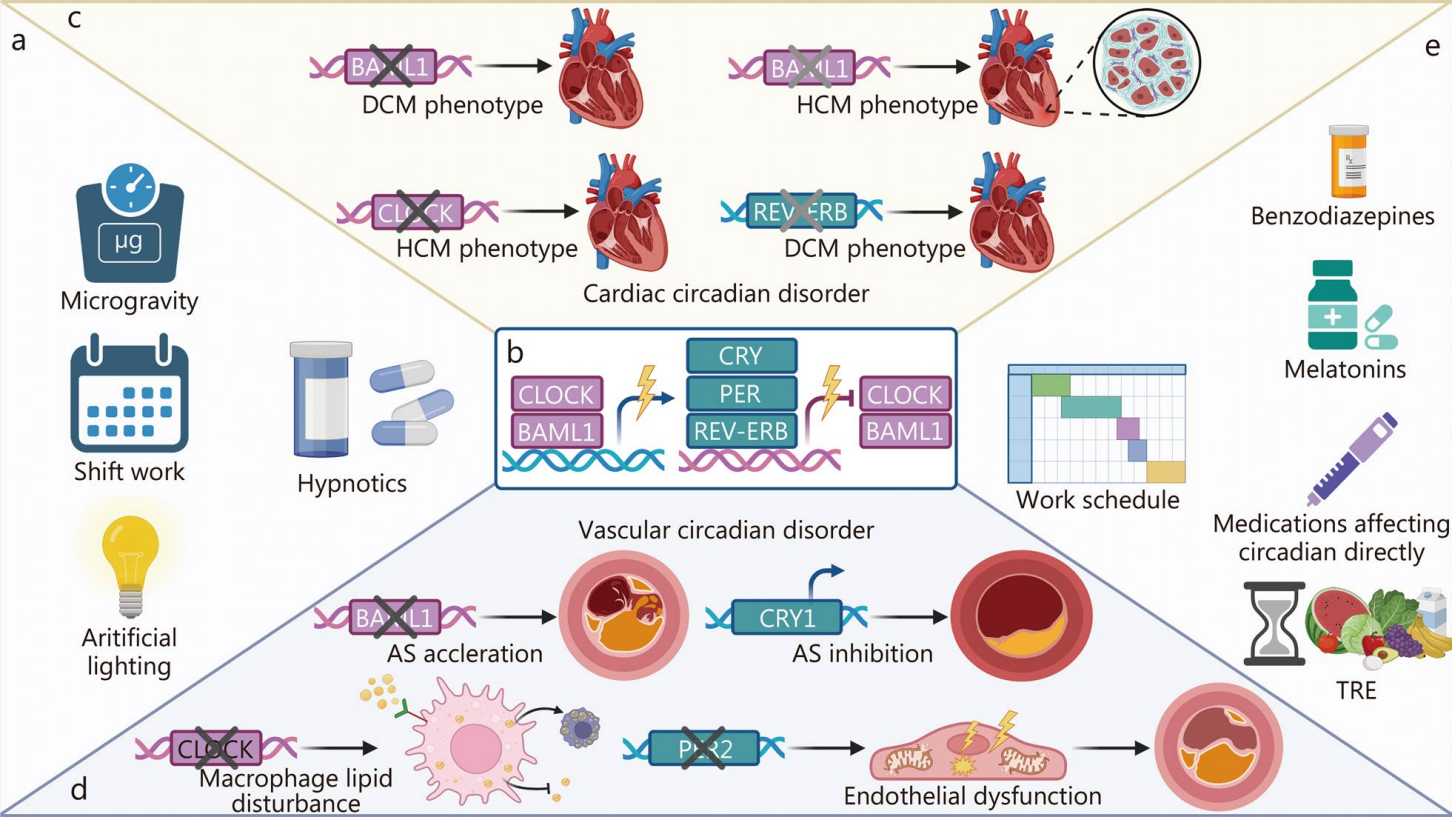
Causes, mechanisms, and possible interventions for cardiovascular alterations induced by circadian rhythm disruption. **a** The broader environmental and lifestyle factors contribute to circadian disruption and subsequent cardiovascular consequences. **b** The transcription-translation feedback loop constructed by core components of the circadian clock can be disrupted during spaceflight. **c** Disruption of *BMAL1*, *CLOCK*, and *REV-ERB* can impair cardiac function and is associated with DCM or HCM phenotypes. **d** Vascular circadian disorders are shown to influence AS acceleration and inhibition, macrophage lipid disturbances, and endothelial dysfunction. **e** The potential countermeasures, including hypnotics, medications directly affecting circadian rhythms, and TRE, can fulfill the comprehensive management strategies to mitigate circadian rhythm disturbances in diverse settings. AS atherosclerosis, BAML1 brain and muscle arnt-like 1, CRY cryptochrome, DCM dilated cardiomyopathy, HCM hypertrophic cardiomyopathy, PER period, TRE time-restricted eating

The human circadian rhythm, naturally synchronized with Earth’s 24-hour light–dark cycle, is vulnerable to disruption by factors such as microgravity, artificial lighting, shift work, and hypnotic use during missions **(**Fig. 3**)**. These disturbances pose a threat to the cardiovascular health of astronauts [162, 163]. Studies on the impacts of long-duration spaceflight on human circadian rhythms are rare. A case report from the Space Station Mir investigated the effects of prolonged spaceflight on the endogenous circadian rhythm and sleep [164]. They found that the function of the endogenous circadian pacemaker, as indicated by subjective alertness rhythms and oral temperature, exhibited time-dependent impairment and compromised sleep. Recent behavioral and transcriptomic studies in both human and animal models have revealed that microgravity might exacerbate the asynchrony of clock gene expression between peripheral tissues, consequently leading to disrupted activity and sleep rhythms [165–168]. Empirical evidence suggests that the space environment can impact the circadian rhythms of blood pressure and HR [169, 170]. Heart rate variability during spaceflight exhibited differences compared to ground missions. Liu et al. [169] documented a notable reduction in HR amplitude, body trunk activity, and rhythmicity among 3 Chinese astronauts during the mission, with 1 experiencing a phase shift in the circadian rhythm of HR. However, the average HR remained unchanged. Long-term observations of 4 Russian astronauts revealed that while the mean systolic blood pressure and HR of astronauts remained stable, their circadian patterns shortened in duration [171]. Notably, Russian astronauts exhibited a significant increase in average systolic blood pressure during sleep. Although isolating the effects of individual factors on the circadian rhythm in space is challenging, overall data underscore the capacity of the space environment to modulate circadian rhythms within the cardiovascular system.

### Mechanisms of cardiac structural and functional impairments

Disruption of core clock genes and disturbances in circadian rhythm have been linked to compromised cardiac structure and function [172]. Global *Bmal1* knockout mice displayed phenotypes of dilated cardiomyopathy, such as myocardial thinning, chamber dilation, and systolic dysfunction [173]. Conversely, specific ablation of *Bmal1* in cardiomyocytes yielded hypertrophic cardiomyopathy characterized by increased cardiomyocyte volume, progressive fibrosis in the interstitial and endocardial regions, and worsening diastolic dysfunction [174]. *Clock* knockout mice showed age-dependent hypertrophic cardiomyopathy, characterized by increased heart weight, myocardial hypertrophy, chamber dilation, compromised contractility, and reduced myogenic responsiveness. These effects may be associated with disrupted diurnal oscillation of *Pten* expression and altered phosphorylation of downstream Akt-glycogen synthase kinase 3β-mitogen-stimulated protein kinase p70 ribosomal protein S6 kinase 1 [175]. *Clock* mutant mice were able to attenuate cardiac hypertrophy, LV remodeling, and diastolic dysfunction through the upregulation of cardiac catalase and glutathione peroxidase induced by obesity and metabolic syndrome [176]. Cardiomyocyte-specific *Rev-erb-α/β* knockout led to progressive deterioration of systolic dysfunction and dilated cardiomyopathy. In the context of obesity with insulin resistance, *Rev-erb* ablation temporarily ameliorated cardiac dysfunction by relieving the suppression of lipid oxidation genes during the light phase. This was mediated by the downregulation of the transcriptional repressor E4 promoter-binding protein 4, thereby augmenting fatty acid oxidation flux [161]. These observations indicate that while disruptions in circadian rhythm may adversely affect cardiac structure and function, enhancing lipid oxidation and antioxidant mechanisms during extended spaceflight could offer protective effects against cardiac alterations.

### Mechanisms of vascular structural and functional deteriorations

The disruption of circadian rhythm is increasingly recognized as a significant factor in vascular injury, potentially accelerating the progression of vascular lesions and elevating the risk of vascular diseases in astronauts [177]. APOE*3-Leiden mice exposed to a simulated shift work schedule, characterized by weekly alternating light–dark cycles, showed marked increases in the size and severity of atherosclerotic lesions. This augmentation was attributed to heightened infiltration of macrophages and the upregulation of inflammatory markers, oxidative stress genes, and chemokines within the vascular wall [178]. These findings underscore the direct influence of circadian rhythm disruption on the acceleration of age-dependent AS, a process seemingly independent of plasma lipid concentrations [178]. In LDLR^−/−^ mice, global *Bmal1* knockout coupled with a high-fat diet accelerated the onset of AS without concurrent changes in plasma total cholesterol and triglyceride levels [179]. Adult *Bmal1* knockout mice demonstrated reduced AS burden and delayed onset when subjected to a high-fat diet [179]. These findings suggest circadian disruption primarily affects the vascular wall locally rather than exerting systemic influences originating from extravascular organs or tissues. The underlying mechanisms for AS acceleration may involve the disconnection of NO synthesis within aortic ECs following *Bmal1* deletion. This process is marked by reduced eNOS cofactor tetrahydrobiopterin levels, leading to eNOS uncoupling, diminished NO production, increased superoxide generation, and subsequent endothelial inflammation [180]. Further insights were obtained from VSMC-specific *Bmal1* knockout mice, in which downregulation of *ARHGDIA* transcription and a weakened interaction between the Ras-related C3 botulinum toxin substrate (RAC1) and BMAL1 were implicated in promoting RAC1-driven VSMC migration. These effects were accompanied by an impairment of antioxidant function through the inhibition of nuclear factor erythroid 2-related factor 2 activity and *Bcl2* transcription. This led to a cascade effect involving monocyte migration, elevated ROS levels, and VSMC apoptosis, thereby intensifying the progression of AS [181]. Furthermore, a relationship between microgravity and intrinsic diurnal oscillation of vasoconstriction has been reported [182]. Simulated microgravity attenuated the diurnal variation of myogenic tone in rat cerebral arteries by altering the circadian regulation of the BMAL1/miR-103/Ca_V_1.2 signaling pathway at the post-transcriptional level. These studies highlight the pivotal roles played by ECs and VSMCs in vascular structure and function impairment induced by circadian rhythm disorder.

Mice with a mutation in the *Clock* gene exhibited hypercholesterolemia when their circadian rhythm was disrupted, which was attributed to enhanced intestinal cholesterol absorption, increased macrophage uptake of modified lipoproteins, and diminished cholesterol efflux. These factors collectively led to accelerated AS [183]. In contrast, *Per2* mutant mice showed endothelial dysfunction, potentially expediting AS through reduced production of NO and vasodilatory prostaglandins, and increased levels of vasoconstrictors derived from cyclooxygenase-1, and Akt-dependent EC aging [184, 185]. Moreover, *Cry1* overexpression attenuates AS by downregulating pro-inflammatory cytokines and leukocyte adhesion molecules related to the Toll-like receptor/NF-κB pathway, while also reducing plasma cholesterol, triglycerides, and low-density lipoprotein cholesterol [186]. These findings indicate the crucial role of an intact circadian rhythm in maintaining vascular structural and functional homeostasis.

### Potential countermeasures targeting circadian rhythm disruption

The significant implications of circadian rhythm disruption highlight the necessity for interventions to prevent astronauts’ insomnia and alleviate the impacts of shift work. The current pharmacological approaches primarily involve benzodiazepines, non-benzodiazepine receptor agonists, melatonin, and melatonin agonists [187]. Benzodiazepines and benzodiazepine receptor agonists directly induce sleep by enhancing the activity of the inhibitory neurotransmitter γ-aminobutyric acid, thereby amplifying inhibitory signals to arousal-promoting cell groups in the brainstem and hypothalamus [188]. Some studies have indicated that these medications may independently lower mortality rates and reduce rehospitalization in HF patients by ameliorating psychosocial risk factors such as insomnia and psychosocial stress [189, 190]. However, side effects including headaches, dizziness, dry mouth, drowsiness, diarrhea, grogginess, and palpitations, as well as the potential of physiological or psychological dependence and tolerance with long-term use restrict their application in astronauts [191], despite their safety and effectiveness for short-term sleep-promotion [192]. Melatonin is considered to be more effective for treating circadian rhythm disruptions or insomnia in individuals with circadian rhythm disorders than sedatives or hypnotics [191]. Therefore, strategic administration of exogenous melatonin, known to induce fatigue and drowsiness, could rectify circadian rhythm disruptions in astronauts [193]. The use of melatonin before sleep has been shown to assist shift workers in adapting to the day-night cycle, extending both daytime and nocturnal sleep and improving overall sleep quality [194]. Further research is needed to investigate the effects of melatonin on sleep latency, insulin resistance, and diurnal blood pressure rhythms in shift workers. Melatonin agonists similarly influence the circadian clock by binding to melatonin receptors and increasing cerebral melatonin levels [191]. Although benzodiazepines and benzodiazepine receptor agonists are more effective than melatonin and its agonists for short- or long-term treatment of insomnia [195], their superior safety, relevance to circadian rhythm disorder, and relatively mild adverse effects may make them more suitable for astronauts [196]. Drugs directly targeting the circadian system have demonstrated promising potential in correcting disruptions to circadian rhythm. SR9009 and SR9011, which are agonists of REV-ERBα, can promote metabolic homeostasis by reducing fat mass and improving hyperglycemia and dyslipidemia in cases of diet-induced obesity [197]. Additionally, SR9011 and SR8278 (a REV-ERBα antagonist) have displayed anxiolytic effects [198, 199], which may help prevent mood vulnerability in shift work settings and anxiety-like mood-associated circadian misalignment [200]. Furthermore, KL001, a compound that stabilizes CRY protein levels leading to extended periods and reduced amplitudes of circadian rhythms, has been found to inhibit glucagon-induced gluconeogenesis [201], potentially improving conditions related to hyperglucagonemia-induced lipotoxicity while delaying the development of insulin resistance and cardiac dysfunction [202].

In addition to pharmacological treatments, improvements in dietary habits and achieving a better work-life balance can help reduce the risk of cardiovascular diseases induced by disruptions in circadian rhythm. Time-restricted eating (TRE), involving the consumption of daily caloric intake within a specific time window followed by fasting without reducing overall caloric intake, has been shown to alleviate circadian rhythm disruption and mitigate cardiovascular aging, thus diminishing the risk of cardiovascular disease in astronauts [203]. A cohort study involving 137 firefighters on 24-hour shifts revealed that a 10-h TRE over 12 weeks significantly reduced very low-density lipoprotein particle size and decreased levels of glycated hemoglobin A1C and diastolic blood pressure, particularly in individuals with elevated baseline cardiometabolic risk. These findings suggest that TRE may improve cardiometabolic health, particularly for populations experiencing circadian rhythm disturbances [204]. The investigation of shift schedules that minimally impact cardiovascular health is of paramount importance. The evidence suggests that limiting consecutive night shifts reduces circadian disruption, and slow rotating shifts exert fewer adverse effects on sleep duration compared to permanent night shifts [205]. Nonetheless, further research is imperative to discern the differential impacts of various shift schedules on cardiovascular health. A proposed schedule aimed at minimizing injury and breast cancer risk may serve as a model for mitigating circadian rhythm disruption in astronauts: (1) ≤ 3 consecutive night shifts, (2) shift intervals ≥ 11 h, and (3) ≤ 9 h of working time [193].

## Leveraging microgravity to promote cardiovascular treatment

hCMs and cardiovascular progenitor cells (CPCs) play a pivotal role in unraveling the intricacies of cardiac pathophysiology, development, and regeneration [206]. The ability to culture these cells in space has been confirmed by various studies [207, 208]. A 3-week study on the ISS observed a significant increase in the expression of genes related to cell proliferation, cardiac differentiation, and cardiac function in hCMs, along with a suppression of ECM regulation genes [207, 209]. After 30 d of exposure to microgravity, neonatal and adult CPCs showed upregulated expression of DNA repair genes and paracrine factors, along with enhanced migratory capacity [208]. Intriguingly, differences in gene expression related to cell proliferation, cardiac regeneration, and development were observed between neonatal and adult CPCs [208]. Neonatal CPCs, in particular, exhibited characteristics indicative of early cardiovascular development and enhanced proliferative capacity, alongside a significant downregulation of the Hippo signaling pathway effector gene *Yap1*. Conversely, adult CPCs showed an upregulation of *Yap1* under microgravity, as supported by subsequent studies [210]. The importance of Yap is highlighted in myocardial regeneration post-infarction, where it promotes cardiomyocyte proliferation and the expression of genes associated with cardiac regeneration, while simultaneously inhibiting cardiac fibroblast activation and trans-differentiation through the enhancement of noncanonical Wnt signaling to prevent cardiac fibrosis [211]. These findings suggest that spaceflight has the potential to address certain challenges inherent in cardiovascular regenerative medicine. Nevertheless, there are still obstacles to overcome, such as the possible decline in enhanced stemness of hCMs or CPCs upon return to Earth and the feasibility of mass-producing personalized hCMs or CPCs with high quality during spaceflight.

## Conclusions

Microgravity, space radiation, and circadian rhythm disruption are recognized as primary risks to cardiovascular health that exacerbate the dangers associated with human space exploration. In the course of spaceflight, there is evidence of accelerated aging-related modifications in the cardiovascular system. After a 6-month mission on the ISS, astronauts exhibit structural and functional changes in their vasculature and heart, including increased thickness and stiffness of blood vessel walls, potentially heightening susceptibility to AS. Numerous aspects necessitate further investigation. Firstly, it is imperative to elucidate the specific mechanisms underlying altered ion channel activity, such as LTCC and RyR, from a structural biology perspective. Subsequently, further research is warranted to comprehensively understand the reversibility of cardiovascular remodeling induced by spaceflight. This necessitates longitudinal studies tracking astronauts’ post-mission to monitor the recovery of cardiovascular function and structure over time; exploration into the molecular mechanisms governing both reversible and irreversible changes for identification of potential therapeutic targets; and advancement in countermeasures development that can be implemented during and after space missions to promote cardiovascular health. Thirdly, there remains a scarcity of information on the molecular connections between the circadian clock and spaceflight-induced circadian disruption leading to cardiovascular malfunction during spaceflight. Therefore, additional evidence is needed in the future. Lastly, given the limitations of diagnostic tools such as echocardiography and photoplethysmography in space, it is crucial to develop more accurate and reliable techniques for monitoring cardiac performance and detecting early signs of cardiovascular dysfunction during space missions. Moreover, continuous monitoring is essential for assessing the potential cardiovascular complications following extended space missions and re-entry to Earth. Careful consideration of these environmental hazards is necessary to accurately evaluate health risks and develop effective countermeasures. The discrepancies between space and simulated terrestrial data underscore the imperative to comprehensively assess the interaction of these complex risk factors. Ongoing and future research efforts are vital for protecting human health during interstellar travel.

## Data Availability

Not applicable.

## Abbreviations

8-oxo-dG: 8-Oxo-deoxy-guanosine
Ω-3: Omega-3 polyunsaturated fatty acids
Akt: Protein kinase B
AS: Atherosclerosis
BFR: Blood flow restriction
BMAL1: Brain and muscle arnt-like 1
BNIP3: B-cell lymphoma 2/adenovirus E1B 19 kD protein-interacting protein
cAMP: Cyclic adenosine monophosphate
CaMKII: Calcium-calmodulin dependent protein kinase II
CHOP: C/EBP-homologous protein
CKIP-1: Casein kinase 2 interacting protein-1
CO: Cardiac output
CPCs: Cardiovascular progenitor cells
Cry: Cryptochrome
CytOx: Cytochrome c oxidase
DPP-4: Dipeptidyl peptidase-4
DVL2: Dishevelled segment polarity protein 2
E/A: Early to late diastolic transmitral flow velocity
ECs: Endothelial cells
ECM: Extracellular matrix
E/E’: Early diastolic transmitral flow velocity to early diastolic mitral annular tissue velocity
EF: Ejection fraction
eNOS: Endothelial NOS
ER: Endoplasmic reticulum
ERK1/2: Extracellular regulated protein kinases 1 and 2
FS: Fractional shortening
GLP-1RA: Glucagon-like peptide 1 receptor agonist
hCMs: Human induced pluripotent stem cell-derived cardiomyocytes
HDAC4: Histone deacetylase 4
HDAC4-NT: N-terminal proteolytic fragment of HDAC4
HF: Heart failure
HLR: Hindlimb reloading
HU: Hindlimb unloading
HUVECs: Human umbilical vein endothelial cells
HZE: Particles high-energy heavy ions
ICAM-1: Intercellular cell adhesion molecule-1
IL: Interleukin
IMT: Intima-media thickness
iNOS: Inducible NOS
ISS: International space station
LEO: Low earth orbit
LET: Linear energy transfer
LTCC: L-type calcium channels
LV: Left ventricle
LVEDD: Left ventricular end-diastolic dimension
LVEDV: Left ventricular end-diastolic volume
LVESD: Left ventricular end-systolic dimension
MDH: Malate dehydrogenase
MEF2C: Myocyte-specific enhancer factor 2C
MMP: Matrix metalloproteinase
NAC: N-acetylcysteine
NASA: National Aeronautics and Space Administration
NCX: Sodium-calcium exchanger
NFATc4: Nuclear factor of activated T-cell c4
NF-κB: Nuclear factor kappa-B
NLRP3: NOD-like receptor thermal protein domain associated protein 3
NO: Nitric oxide
NOS: Nitric oxide synthase
NOX: NADPH oxidase
NR: Nicotinamide riboside
PER: Period
PINK1: PTEN-induced putative kinase 1
PKA: Protein kinase A
PP2A: Protein phosphatase 2A
RAC1: Ras-related C3 botulinum toxin substrate
ROS: Reactive oxygen species
RyR2: Ryanodine receptor 2
SERCA: SR Ca^2+^-ATPase
SGLT2: Sodium-glucose cotransporter-2
SR: Sarcoplasmic reticulum
SV: Stroke volume
TRE: Time-restricted eating
TS: Tail suspension
VCAM-1: Vascular cellular adhesion molecule-1
VSMCs: Vascular smooth muscle cells
WWP1: WW domain-containing E3 ubiquitin protein ligase 1
